# Influence of housing retaining materials on fracture resistance of zirconia-reinforced implant overdentures: an *in vitro* study

**DOI:** 10.3389/fdmed.2025.1692255

**Published:** 2026-01-22

**Authors:** Shradha Bhutoria, Thilak Shetty, Shobha J. Rodrigues, Umesh Y. Pai, Sharon Saldanha, Mahesh M, Ann Sales, Sandipan Mukherjee, Vignesh Kamath, Prashant Bajantri

**Affiliations:** Department of Prosthodontics, Manipal College of Dental Sciences Mangalore, Manipal Academy of Higher Education, Manipal, India

**Keywords:** flexural strength, implant supported overdenture, PMMA, SEM, surface characterisation

## Abstract

**Aim:**

To evaluate the influence of zirconium oxide (ZrO₂) nanoparticle reinforcement and housing retaining material on the flexural performance of implant-supported overdenture acrylic resin bases.

**Materials and methods:**

Thirty-six standardized bar-shaped heat-cured polymethylmethacrylate (PMMA) specimens were fabricated in accordance with ISO 20795-1 and divided into six groups (*n* = 6) based on ZrO₂ nanoparticle concentration (0%, 3%, and 7% by weight) and housing retaining material (autopolymerising PMMA or UfiGel Hard). Specimens were subjected to thermocycling to simulate oral aging and tested under three-point bending to determine flexural strength. Surface characteristics were qualitatively assessed using scanning electron microscopy (SEM) and atomic force microscopy (AFM). Data were analyzed using one-way ANOVA with Tukey's *post hoc* test (*α* = 0.05).

**Results:**

ZrO₂ nanoparticle reinforcement significantly enhanced flexural strength across both retaining materials. Specimens containing 7 wt% ZrO₂ demonstrated approximately 75%–90% higher flexural strength compared with non-reinforced controls, depending on the retaining material employed. All reinforced groups exceeded the minimum flexural strength requirement of 65 MPa specified by ISO 20795-1 for denture base polymers. Autopolymerising PMMA consistently exhibited higher flexural strength than UfiGel Hard. SEM revealed differences in nanoparticle dispersion with increasing concentration, while AFM demonstrated increased surface roughness at higher ZrO₂ levels. These improvements suggest enhanced resistance to housing-related fracture, a common clinical complication in implant-supported overdentures.

**Conclusion:**

Zirconium oxide nanoparticle reinforcement significantly enhanced the flexural strength of implant-supported overdenture acrylic resin bases, with superior performance observed when autopolymerising PMMA was used as the housing retaining material. These findings suggest a material-based approach to improving overdenture base fracture resistance; however, clinical extrapolation should be approached with caution, and further studies incorporating fatigue loading and long-term aging are warranted.

## 1 Introduction

Implant-supported overdentures (IODs) are a well-established treatment modality for edentulous patients, offering improved retention, masticatory efficiency, patient satisfaction, and preservation of residual alveolar bone when compared with conventional complete dentures (1–4). The retention of IODs is commonly achieved using attachment systems based on a matrix–patrix design, wherein a metal housing is incorporated into the denture base during chairside pickup procedures (5).

**Figure 1:**
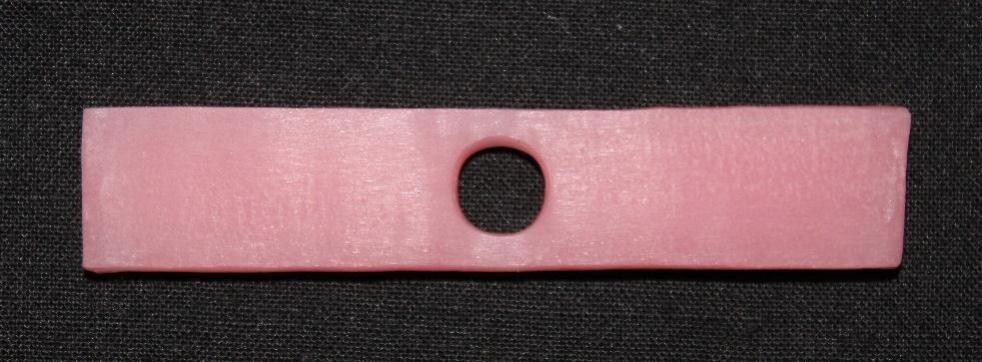
Wax model acrylized with heat cure polymethylmethacrylate.

**Figure 2:**
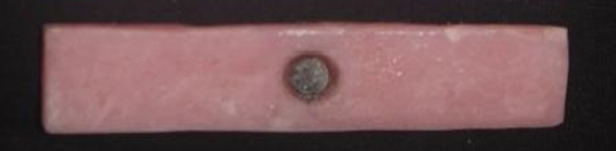
Final acrylized specimen with implant housing retained by housing retaining.

Although these attachment systems enhance prosthesis stability, the incorporation of housings frequently results in localized reduction in denture base thickness, creating a biomechanically vulnerable region that is prone to stress concentration and fracture under functional loading (6, 7).

Most investigations evaluating denture base reinforcement have primarily focused on bulk material properties, with limited attention directed toward the clinically critical housing–resin interface in implant-supported overdentures. This region is subjected to complex tensile, compressive, and shear stresses during mastication and parafunctional activity, and differences in housing retaining material stiffness, bonding behavior, and polymerization characteristics may significantly influence stress distribution and fracture resistance (7–10). Consequently, improving the mechanical performance of the denture base material surrounding attachment housing remains a clinically relevant challenge. A variety of retaining materials are employed for housing pickup procedures, including autopolymerising polymethyl methacrylate (PMMA), hard chairside relining materials such as UfiGel Hard, and, less commonly, composite- or pattern resin–based materials (7, 11, 12).

Autopolymerising PMMA offers chemical compatibility with heat-cured PMMA denture bases and forms a relatively rigid interface, whereas UfiGel Hard is designed to exhibit greater resilience and stress-absorbing capacity (7–9). Despite their widespread clinical use, evidence comparing the interaction of these retaining materials with reinforced denture base resins remains limited, particularly in the context of nanoparticle-modified PMMA.

To enhance the mechanical properties of PMMA denture base resins, several reinforcement strategies have been proposed, including metal meshes, fibers, cross-linking agents, and, more recently, nanoparticle incorporation (13–15).

Among these, zirconium oxide (ZrO₂) nanoparticles have received considerable attention due to their favorable biocompatibility, esthetic compatibility, high fracture toughness, and ability to improve flexural and impact strength through dispersion strengthening and transformation toughening mechanisms (14, 16, 17).

Experimental studies have demonstrated that low to moderate concentrations of ZrO₂ nanoparticles (typically between 1 and 7 wt%) can significantly enhance the mechanical performance of PMMA denture base materials (13, 16–18).

However, the literature reports contradictory findings regarding the effect of higher nanoparticle concentrations on resin performance. While some studies report continued improvement in flexural strength with increasing ZrO₂ content (16–18), others have demonstrated that excessive nanoparticle loading may promote agglomeration within the resin matrix, leading to stress concentration sites, increased surface roughness, and compromised mechanical properties (14, 17, 18). While nanoparticle reinforcement has shown promise, inconsistent findings regarding agglomeration and stress concentration highlight the need to evaluate reinforcement strategies within clinically relevant prosthetic interfaces.Importantly, most existing studies have evaluated nanoparticle-reinforced PMMA in isolation, without considering the influence of different housing retaining materials on the mechanical behavior of implant overdenture bases. Given that the housing–resin interface represents a critical site of stress transmission and fracture initiation, understanding the combined effect of denture base reinforcement and retaining material selection is essential for improving clinical longevity.

Therefore, the present *in vitro* study aimed to evaluate the effect of zirconium oxide nanoparticle reinforcement of heat-cured PMMA denture base resin on the flexural strength of implant-supported overdenture bases using two commonly employed and mechanically distinct housing retaining materials: autopolymerising PMMA and UfiGel Hard. These materials were selected to represent rigid and resilient clinical pickup materials, thereby allowing isolation of the influence of housing material stiffness on reinforced denture base performance.

The null hypothesis was that the incorporation of zirconium oxide nanoparticles would not significantly affect the flexural strength of implant-supported overdenture acrylic resin bases, irrespective of the housing retaining material used.

## 2 Material and methods

### 2.1 Study design and specimen grouping

This *in vitro* experimental study evaluated the effect of zirconium oxide (ZrO₂) nanoparticle reinforcement and housing retaining material on the flexural strength of implant-supported overdenture acrylic resin bases. A total of 36 specimens were fabricated and divided into six groups (*n* = 6) based on two variables based on a previous study (13):

ZrO₂ nanoparticle concentration (0%, 3%, and 7% by weight), and
Housing retaining material (autopolymerising PMMA or UfiGel Hard).

Non-reinforced specimens served as control groups for each retaining material (Table 1).

**Table 1:** The groups in the study.

Reinforcement	Autopolymerising PMMA	UfiGel hard
No reinforcement	Group 1	Group 4
3% ZrO₂ NPs	Group 2	Group 5
7% ZrO₂ NPs	Group 3	Group 6

### 2.2 Specimen fabrication

Rectangular bar-shaped specimens (64 × 10 × 3 mm) were fabricated using heat-cured PMMA denture base resin (Figures 1, 2) in accordance with ISO 20795-1 specifications for flexural strength testing (16) and following manufacturer's instructions to reduce residual monomer content. A standardized cylindrical cavity (8 mm diameter, 3 mm depth) was created at the center of each specimen to simulate housing placement. Commercially available metal housings (Genesis Implant System, Kerala; 4.05 × 2.9 mm) were positioned within the cavity.

### 2.3 Zirconia nanoparticle incorporation

Silane-treated zirconium oxide nanoparticles (average particle size ≈ 40 nm) were weighed to achieve 3 wt% and 7 wt% concentrations and incorporated into the PMMA powder using manual blending followed by mechanical mixing to promote uniform distribution. The reinforced powder was then polymerized with monomer following the manufacturer's instructions. No quantitative analysis of nanoparticle dispersion was performed; dispersion and agglomeration patterns were evaluated qualitatively using scanning electron microscopy (SEM).

### 2.4 Housing pickup procedure

Housing pickup was performed using either autopolymerising PMMA or UfiGel Hard, according to the assigned group. No additional bonding primers or surface conditioners were used, as the study aimed to assess the intrinsic mechanical interaction between commonly used clinical retaining materials and nanoparticle-reinforced PMMA under standardized conditions. This approach was selected to reflect routine chairside clinical practice. Residual monomer content was not evaluated and is acknowledged as a study limitation.

### 2.5 Finishing and polishing

All specimen surfaces, except those designated for SEM and AFM analysis, were finished using progressively finer silicon carbide abrasive papers (320, 600, 800, and 1,200 grit) under continuous water irrigation. Polishing was completed using pumice and a soft rag wheel. Care was taken to avoid alteration of the internal housing–resin interface.

### 2.6 Thermocycling

All specimens were subjected to 5,000 thermocycles between 5°C and 55°C with a dwell time of 30 s to simulate oral thermal aging prior to mechanical testing (8, 9).

### 2.7 Flexural strength testing

Flexural strength was evaluated using a three-point bending test on a universal testing machine at a crosshead speed of 5 mm/min (7). The load was applied at the midpoint of each specimen until fracture. Flexural strength values were calculated in megapascals (MPa) using the standard formula specified in ISO 20795-1.

### 2.8 Surface characterization

Surface morphology and nanoparticle dispersion was assessed qualitatively using SEM to identify clustering patterns relevant to fracture behavior, consistent with prior exploratory studies. Atomic force microscopy (AFM) was performed on one representative specimen per group to assess nanoscale surface topography (19). AFM was employed as a qualitative surface characterization tool, as it examines localized regions and requires specimen sectioning that precludes subsequent mechanical testing. AFM findings were therefore interpreted descriptively and not subjected to statistical analysis.

### 2.8 Statistical analysis

Data normality was confirmed using the Shapiro–Wilk test. Flexural strength values were analyzed using one-way analysis of variance (ANOVA) followed by Tukey's *post hoc* test for pairwise comparisons. Statistical analysis focused on hypothesis testing rather than estimation statistics due to the exploratory nature of the study and limited subgroup size. The level of significance was set at ***α*** **=** **0.05**. All analyses were performed using SPSS software (version 26.0; IBM Corp).

## 3 Results

All specimens were successfully tested, and no pre-test failures occurred. Flexural strength differed significantly according to retaining material type and zirconium oxide (ZrO₂) nanoparticle concentration.

**Figure 7:**
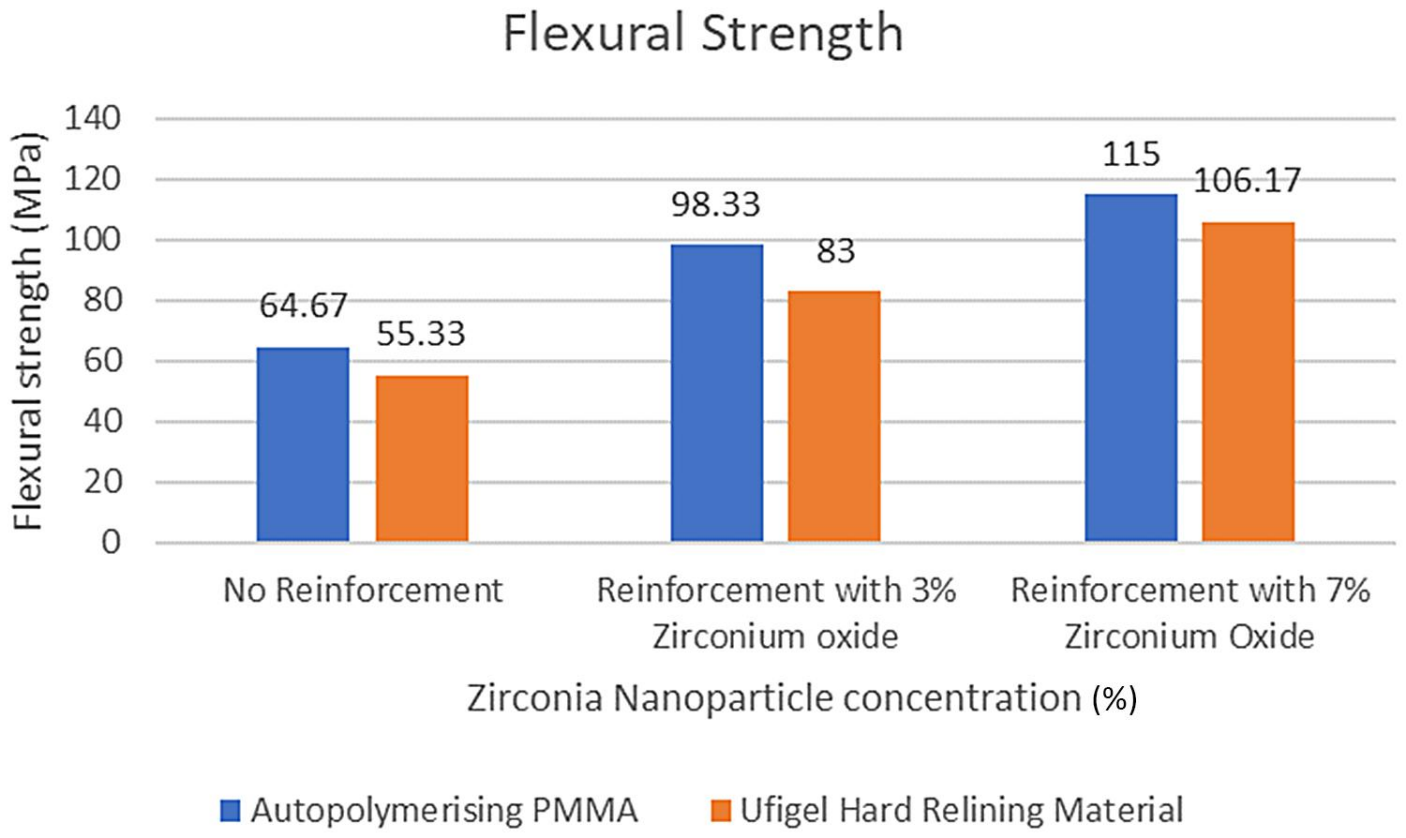
Comparison of flexural strength between reinforcement groups.

**Figure 8:**
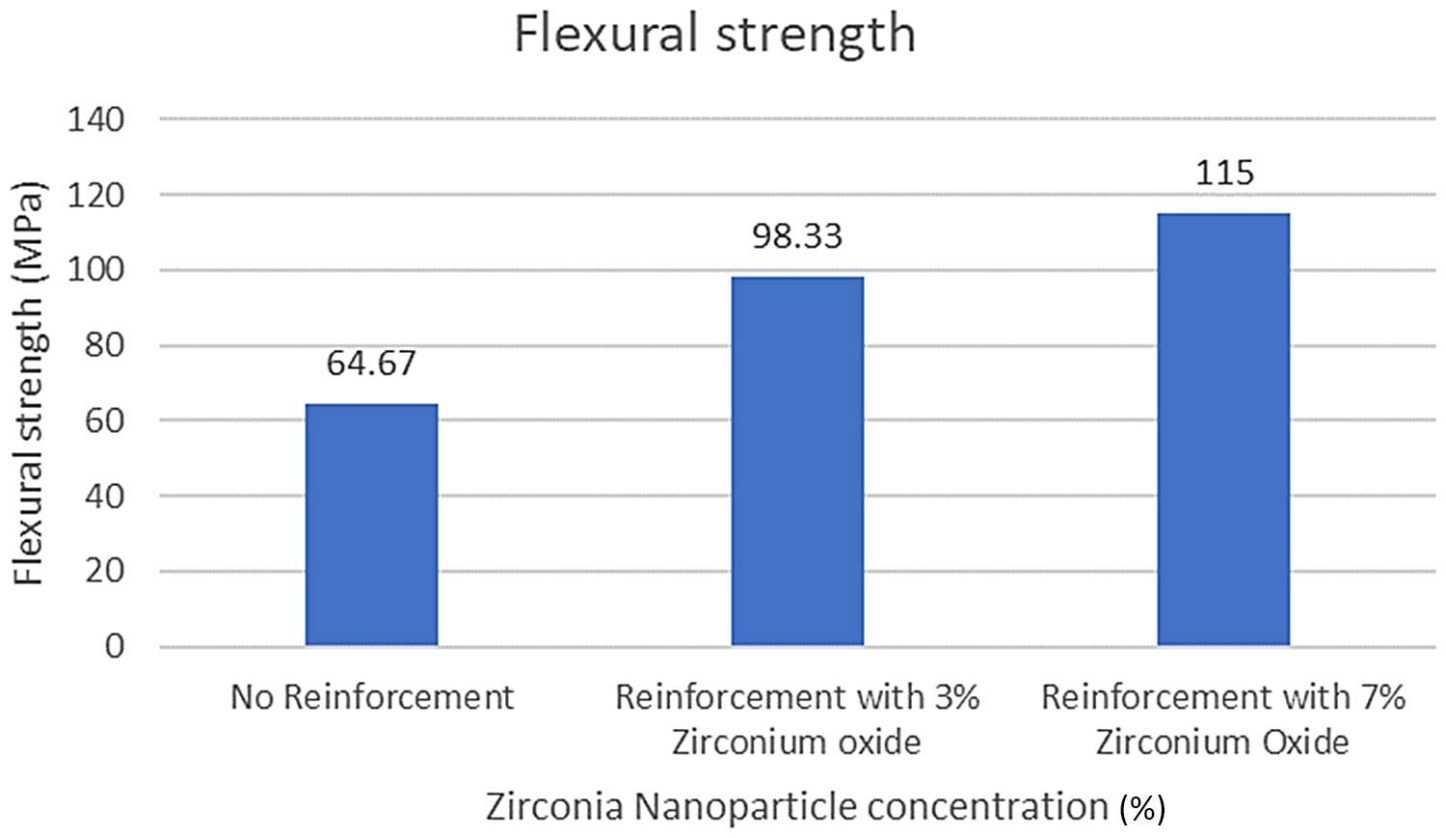
Flexural strength (comparison between reinforcement groups within autopolymerising PMMA housing retaining material).

**Figure 9:**
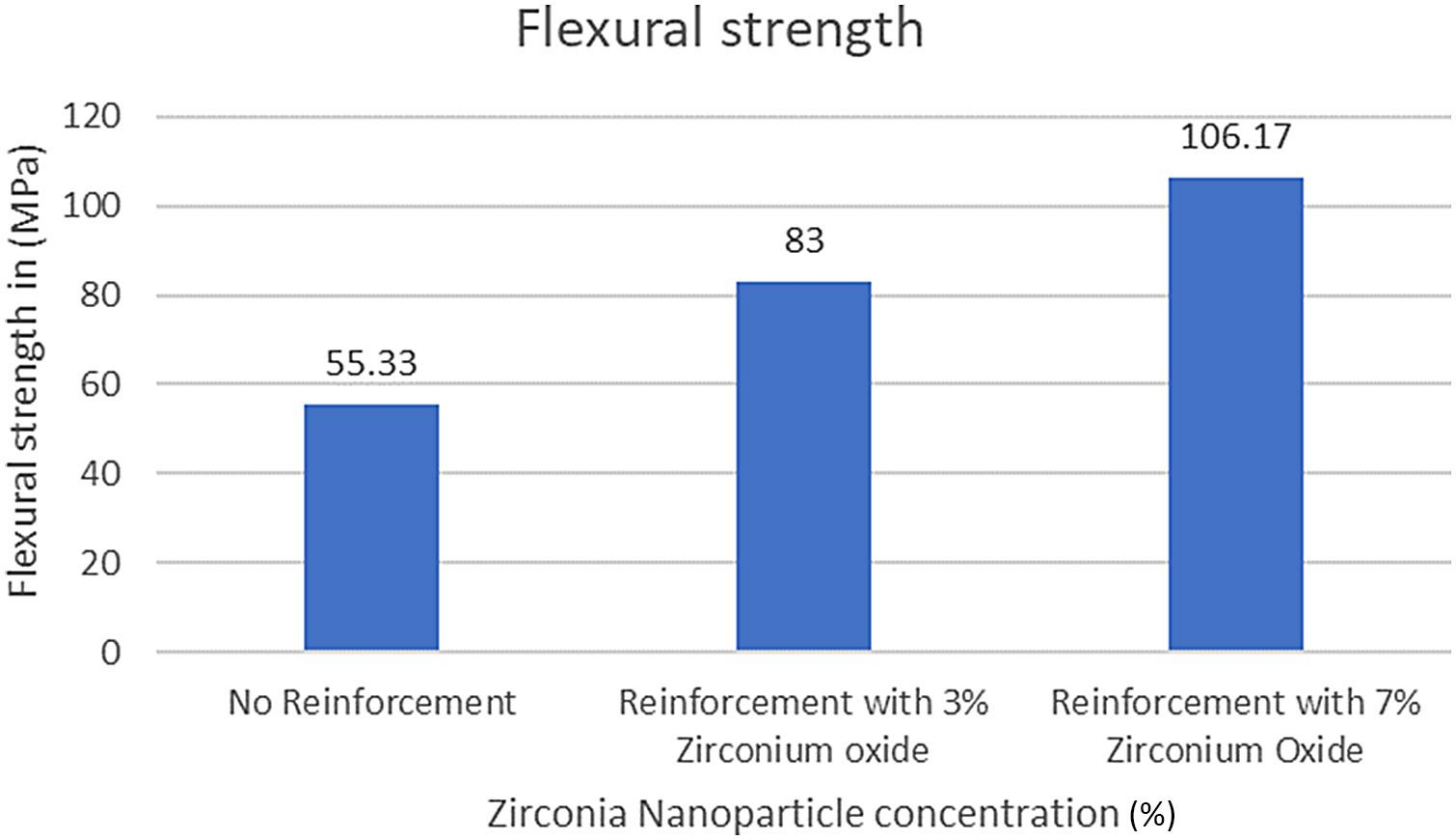
Flexural strength (comparison between reinforcement groups within UfiGel hard housing retaining material).

Results are presented in Tables 2–5; Figures 7–9 as comparative trends to highlight the influence of nanoparticle concentration and housing retaining material on flexural strength.

**Table 2:** Flexural strength (comparison between housing retaining materials).

	Autopolymerising PMMA (*n* = 6)	Ufigel hard relining material (*n* = 6)	t	*P* value
	Mean ± sd	Mean ± sd		
Control	64.67 ± 6.38	55.33 ± 3.67	3.107	**0**.**011**
Reinforcement with 3% Zirconium oxide	98.33 ± 5.85	83 ± 3.03	5.697	**<0**.**001**
Reinforcement with 7% Zirconium Oxide	115 ± 4.73	106.17 ± 2.32	4.106	**0**.**002**

Significant values are indicated in bold.

**Table 3:** Flexural strength (comparison between reinforcement groups within autopolymerising PMMA housing retaining material).

	Control (*n* = 6)	Reinforcement with 3% Zirconium oxide (*n* = 6)	Reinforcement with 7% Zirconium Oxide (*n* = 6)	One way ANOVA		*Post hoc* Tukey test		
				F value (* = welch test)	*P* value	No Reinforcement vs. Reinforcement with 3% Zirconium oxide difference (*p* value)	No Reinforcement vs. Reinforcement with 7% Zirconium Oxide difference (*p* value)	Reinforcement with 3% Zirconium oxide vs. Reinforcement with 7% Zirconium Oxide difference (*p* value)
Flexural strength	55.33 ± 3.67	83 ± 3.03	106.17 ± 2.32	415.88	**<0.001**	−27.67 (<0.001)	−50.83 (<0.001)	−23.17 (<0.001)

Significant values are indicated in bold.

**Table 4:** Flexural strength (comparison between reinforcement groups within UfiGel hard housing retaining material).

	Control (*n* = 6)	Reinforcement with 3% Zirconium oxide (*n* = 6)	Reinforcement with 7% Zirconium Oxide (*n* = 6)	One way ANOVA		*Post hoc* Tukey test		
				*F* value (* = welch test)	*P* value	No Reinforcement vs. Reinforcement with 3% Zirconium oxide difference (*p* value)	No Reinforcement vs. Reinforcement with 7% Zirconium Oxide difference (*p* value)	Reinforcement with 3% Zirconium oxide vs. Reinforcement with 7% Zirconium Oxide difference (*p* value)
Flexural strength	64.67 ± 6.38	98.33 ± 5.85	115 ± 4.73	121.582	**<0.001**	−33.67 (<0.001)	−50.33 (<0.001)	−16.67 (<0.001)

Significant values are indicated in bold.

**Table 5:** Roughness values of all groups at 5 µm (Ra, Rq, Rz).

Type of roughness	Control Group – (In nm)	3% reinforcement (In nm)	7% Reinforcement (In nm)
Arithmetic mean roughness (Ra)	6.46	44.1	**72.5**
Root mean square roughness (Rq)	9.26	64.2	**92.7**
Maximum peak value (Rz)	**188**	**463**	**748**

Significant values are indicated in bold.

### 3.1 Flexural strength

Flexural strength values for all experimental groups are summarized in Tables 2–4. Incorporation of zirconia nanoparticles resulted in a progressive and consistent increase in flexural strength for both housing retaining materials when compared with non-reinforced controls.

Specimens reinforced with 3% zirconia nanoparticles demonstrated a marked improvement in flexural strength relative to their respective control groups, while 7% reinforcement produced the highest strength values across both retaining materials. Notably, all zirconia-reinforced groups exceeded the minimum flexural strength requirement of 65 MPa specified by ISO 20795-1, whereas non-reinforced specimens retained with UfiGel Hard approached this threshold.

Across all reinforcement levels, specimens retained using autopolymerising PMMA exhibited significantly higher flexural strength than those retained with UfiGel Hard. This trend indicates that the stiffness and bonding characteristics of the housing retaining material influence the load-bearing capacity of the overdenture base, particularly under bending forces.

**Figure 3:**
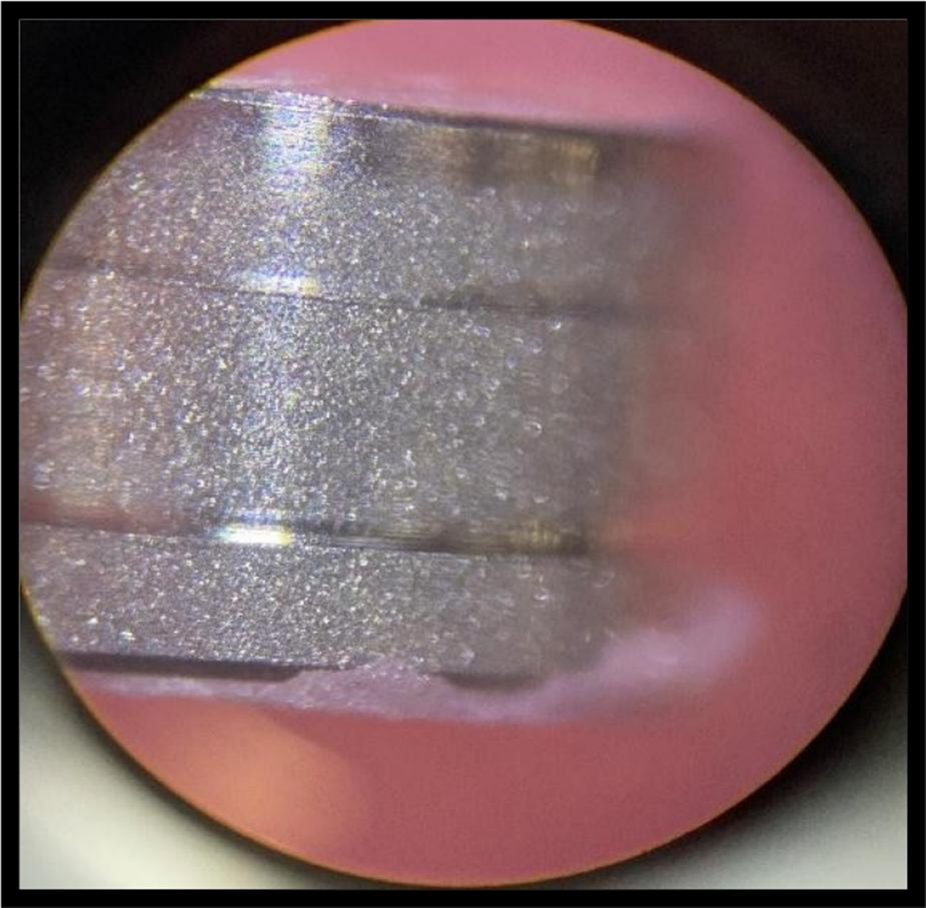
Adhesive failure seen in all groups. (In stereomicroscope under 40X – control group).

Failure analysis revealed that adhesive fractures predominantly initiated at the housing–resin interface, regardless of nanoparticle concentration (Figure 3). This observation underscores the clinical relevance of housing material selection, as this region represents a critical stress concentration zone in implant-supported overdentures.

### 3.2 Analysis within retaining material groups

When flexural strength values were analyzed within each zirconia concentration, autopolymerising PMMA consistently demonstrated superior performance compared with UfiGel Hard. The difference was most pronounced in the reinforced groups, suggesting that rigid housing materials may better transmit and distribute stresses within zirconia-reinforced PMMA matrices, whereas more resilient materials may permit localized deformation under load.

These findings are reflected in the intergroup comparisons shown in Tables 3, 4, where statistically significant differences were observed between retaining materials at all reinforcement levels.

### 3.3 Surface characterization

#### 3.3.1 Scanning electron microscopy (SEM)

**Figure 4:**
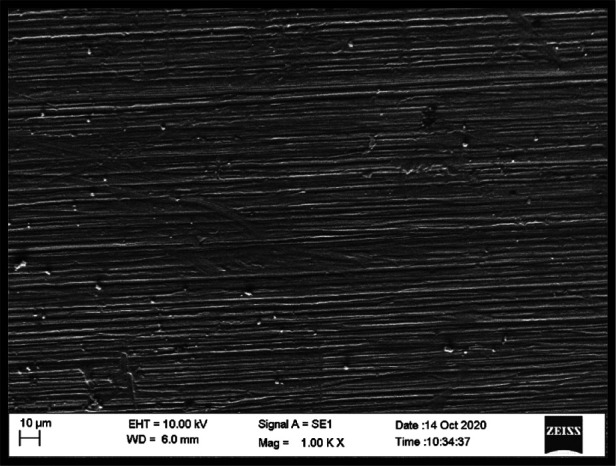
SEM image (1000X) – control group.

**Figure 5:**
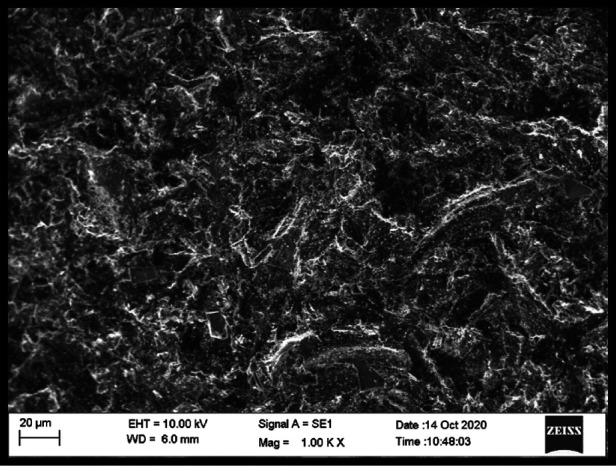
SEM image (1000X) – 3% zirconium oxide reinforcement.

**Figure 6:**
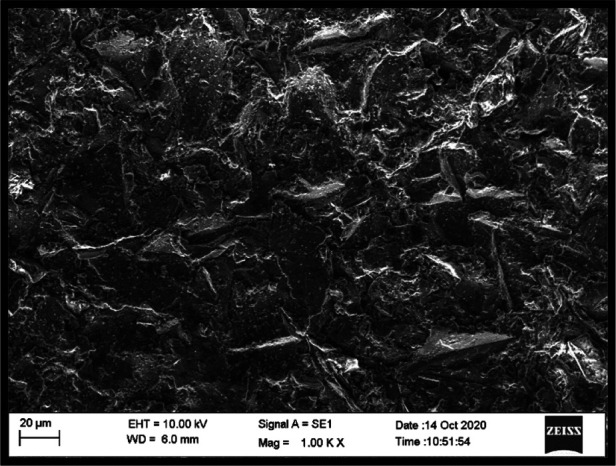
SEM image (1000X) - 7% zirconium oxide reinforcement.

SEM evaluation revealed distinct differences in microstructural features between reinforced and non-reinforced specimens. Control specimens exhibited a relatively homogeneous PMMA matrix with smooth fracture surfaces. In contrast, zirconia-reinforced specimens demonstrated embedded nanoparticle clusters within the resin matrix, with more evident clustering observed at the 7% concentration (Figures 4–6).

Differences were also noted between housing retaining materials. Specimens retained with autopolymerising PMMA exhibited closer adaptation and fewer interfacial voids at the housing–resin junction compared with those retained using UfiGel Hard, which showed irregular interfaces and localized gaps. These interfacial features may partially explain the lower flexural strength values observed in UfiGel Hard groups.

Nanoparticle dispersion was assessed qualitatively, and while general distribution appeared acceptable, localized agglomeration was observed at higher concentrations.

### 3.4 Atomic force microscopy (AFM)

AFM analysis demonstrated a progressive increase in surface roughness with increasing zirconia nanoparticle concentration (Table 5). Non-reinforced specimens exhibited relatively smooth surface topography, whereas 7% zirconia-reinforced specimens showed pronounced nanoscale peaks and valleys.

Surface roughness values were influenced by the type of housing retaining material. Specimens retained with UfiGel Hard exhibited slightly higher surface irregularities adjacent to the housing region compared with those retained with autopolymerising PMMA. Increased surface roughness at higher nanoparticle concentrations may enhance mechanical interlocking within the resin matrix but may also have implications for plaque retention and long-term surface wear.

As AFM was performed on a single representative specimen per group, these observations were interpreted descriptively rather than statistically. Its findings were used to support microstructural interpretation rather than as a determinant of mechanical performance.

### 3.5 Statistical comparison (*post-hoc* analysis)

Crucially, the interaction between the retaining material and the nanoparticle concentration was also statistically significant (*p* < 0.001), confirming that the degree of strengthening achieved by the ZrO₂NPs differs based on the retaining material used.

PMMA housing showed a large initial increase from 0% to 3% reinforcement (33.66 MPa), whereas the UfiGel Hard housing showed a greater increase between the 3% and 7% concentration levels (23.17 MPa), indicating the materials react differently to ZrO₂NPs loading across the concentration range.

## 4 Discussion

The present *in-vitro* study evaluated the effect of zirconia nanoparticle reinforcement and housing retaining material on the flexural strength of implant-supported overdenture (IOD) acrylic resin bases. Based on the statistically significant improvement in flexural strength observed with zirconium nanoparticle reinforcement and the influence of housing retaining material, the null hypothesis was rejected. This discussion focuses on mechanistic explanations, surface–structure relationships, and clinical relevance within experimental constraints. The present findings represent baseline material behavior under static loading, which precedes fatigue testing in material validation pathways.

### 4.1 Influence of zirconia nanoparticle reinforcement on flexural strength

The observed improvement in flexural strength with zirconia nanoparticle incorporation can be attributed to several reinforcing mechanisms. Zirconia nanoparticles act as stress transfer mediators within the PMMA matrix, restricting crack initiation and propagation under bending loads. The progressive increase in strength with higher nanoparticle concentration suggests enhanced load distribution within the resin matrix, although this effect may be counterbalanced by particle agglomeration at higher concentrations.

SEM observations support this explanation by demonstrating the presence of embedded zirconia particles within the PMMA matrix. While dispersion appeared generally acceptable, localized clustering was evident at higher concentrations, consistent with reports that excessive nanoparticle loading may introduce stress concentration zones. These findings help reconcile contradictory reports in literature, where improvements in mechanical properties are observed up to an optimal nanoparticle concentration, beyond which agglomeration may negate reinforcing benefits (10, 20–22).

### 4.2 Comparison between housing retaining materials

When flexural strength values were analyzed within each zirconia concentration, autopolymerising PMMA consistently demonstrated superior performance compared with UfiGel Hard. The difference was most pronounced in the reinforced groups, suggesting that rigid housing materials may better transmit and distribute stresses within zirconia-reinforced PMMA matrices, whereas more resilient materials may permit localized deformation under load.

These findings are reflected in the intergroup comparisons shown in Tables 2–4 where statistically significant differences were observed between retaining materials at all reinforcement levels.

SEM analysis revealedspecimens retained with autopolymerising PMMA exhibited closer adaptation and fewer interfacial voids at the housing–resin junction compared with those retained using UfiGel Hard, which showed irregular interfaces and localized gaps. Autopolymerising PMMA, owing to its higher modulus of elasticity and chemical compatibility with the heat-cured PMMA base, creates a superior cohesive bond at the interface and may facilitate more effective stress transfer across the housing interface. Autopolymerising PMMA uses an MMA monomer-based liquid, which allows for swelling and subsequent copolymerization with the surrounding heat-cured PMMA denture base (13). In contrast, UfiGel Hard, a silicone based PMMA, is formulated to provide a degree of resilience, which may reduce localized stress transmission but also lower the overall resistance of the system to bending forces. It also relies primarily on a manufacturer-supplied adhesive primer and mechanical interlocking for retention, resulting in a less intimate and weaker bond to the PMMA base.

Another critical finding from the study was observation of adhesive failure at the interface between the housing retaining material and the denture base in all specimens. This confirms that despite the significant increase in the bulk strength of the reinforced PMMA matrix, the interface remains the definitive weakest link in the implant-supported overdenture complex. This emphasizes that future research must focus not only on reinforcing the bulk material but also on improving the bond strength at this critical junction, perhaps through surface treatments of the housing material or better adhesive systems. Increased surface roughness at higher nanoparticle concentration as observed in AFM may contribute to mechanical interlocking, its clinical relevance lies primarily in hygiene maintenance rather than fracture resistance.

Since it is in the intaglio surface of the denture attention must be drawn to clean this surface too (10). From a clinical standpoint, high-gloss polishing of reinforced dentures may be advisable to mitigate plaque accumulation. Although implant-supported overdentures demonstrate high implant survival rates, prosthetic complications remain prevalent in long-term clinical follow-up (2–4). Fracture of the acrylic denture base, particularly in the region surrounding attachment housings, has been consistently reported as one of the most common causes of maintenance and repair. Long-term cohort studies and systematic reviews indicate that such failures negatively affect patient satisfaction, increase chairside maintenance, and compromise prosthesis longevity. Therefore, material-based strategies aimed at reinforcing the denture base in clinically vulnerable regions may have meaningful implications for long-term overdenture performance and patient-centered outcomes.

The present *in-vitro* findings suggest that zirconia nanoparticle reinforcement may enhance the baseline flexural resistance of overdenture base resins, potentially contributing to improved resistance against fracture initiation under functional loading. Nevertheless, these results should be interpreted cautiously. The absence of fatigue testing, long-term cyclic loading, and clinical aging protocols limits direct extrapolation to long-term clinical performance. Accordingly, while zirconia nanoparticle reinforcement demonstrates promise as a material-level strategy, prospective clinical studies and fatigue-based investigations are necessary before definitive clinical recommendations can be made.

### 4.3 Study limitations, future scope and clinical considerations

Several limitations of this study should be acknowledged. The investigation was limited to static flexural strength testing and did not include fatigue or cyclic loading protocols, which are critical for simulating long-term clinical function (23). Additionally, thermocycling was restricted to a limited number of cycles and may not fully represent long-term oral aging. Only two housing retaining materials and a single denture base resin system were evaluated, which limits the generalizability of the findings to other materials and attachment systems.

Given these limitations and the *in-vitro* design, clinical recommendations should be interpreted cautiously. The findings should be viewed as indicative of material behavior under controlled conditions rather than direct predictors of long-term clinical performance. Further studies incorporating fatigue loading, push out bond strength extended aging protocols, and a broader range of resin and retaining materials are required before definitive clinical conclusions can be drawn (24).

## 5 Conclusion

Within the limitations of this *in-vitro* study, zirconia oxide nanoparticle reinforcement significantly enhanced the flexural strength of implant-supported overdenture acrylic resin bases under static loading conditions. Increasing nanoparticle concentration resulted in a progressive improvement in flexural strength, with reinforced specimens consistently exceeding the minimum flexural strength requirement specified by ISO 20795-1.

The choice of housing retaining material influenced mechanical performance, with autopolymerising PMMA demonstrating higher flexural strength values compared with UfiGel Hard. These findings suggest that both denture base reinforcement and housing retaining material selection play an important role in the mechanical behavior of implant-supported overdenture bases.

However, as the study was conducted under controlled *in-vitro* conditions without fatigue loading or long-term aging protocols, the results should be interpreted as indicative of material performance rather than direct predictors of clinical longevity. Further investigations incorporating cyclic loading, extended thermomechanical aging, and a wider range of denture base and retaining materials are required to validate the clinical relevance of these findings.

## Data Availability

The original contributions presented in the study are included in the article/Supplementary Material, further inquiries can be directed to the corresponding authors.
